# Tracing Evolution Through Protein Structures: Nature Captured in a Few Thousand Folds

**DOI:** 10.3389/fmolb.2021.668184

**Published:** 2021-05-10

**Authors:** Nicola Bordin, Ian Sillitoe, Jonathan G. Lees, Christine Orengo

**Affiliations:** ^1^ Institute of Structural and Molecular Biology, University College London, London, United Kingdom; ^2^ Department of Biological and Medical Sciences, Faculty of Health and Life Sciences, Oxford Brookes University, Oxford, United Kingdom

**Keywords:** bioinformatics and computational biology, protein structural and functional analysis, structural bioinformatics, protein evolution, protein structure classification

## Abstract

This article is dedicated to the memory of Cyrus Chothia, who was a leading light in the world of protein structure evolution. His elegant analyses of protein families and their mechanisms of structural and functional evolution provided important evolutionary and biological insights and firmly established the value of structural perspectives. He was a mentor and supervisor to many other leading scientists who continued his quest to characterise structure and function space. He was also a generous and supportive colleague to those applying different approaches. In this article we review some of his accomplishments and the history of protein structure classifications, particularly SCOP and CATH. We also highlight some of the evolutionary insights these two classifications have brought. Finally, we discuss how the expansion and integration of protein sequence data into these structural families helps reveal the dark matter of function space and can inform the emergence of novel functions in Metazoa. Since we cover 25 years of structural classification, it has not been feasible to review all structure based evolutionary studies and hence we focus mainly on those undertaken by the SCOP and CATH groups and their collaborators.

## The Early Days–Chothia the Pioneer

Protein structures have helped us see more clearly into the evolutionary past. Cyrus Chothia, to whom this special issue is dedicated, was an early pioneer on these journeys and remained a leading figure throughout his life. As structures accumulated in the Protein Data Bank (PDB) from the early 1970s onwards, he was one of the first to realise the value of comparing them to capture their differences and thereby understand the mechanisms by which proteins evolve. In a similar timeframe i.e. the late 70s and early 80s, another early pioneer in the protein world, Margaret Dayhoff, was also cataloging evolutionary changes by considering the substitutions, insertions and deletions in the amino acid residues that can occur in the protein’s polypeptide chain. By linking these data, we can see how genetic variations translate to structural and ultimately functional impacts. Over the last two decades the explosion in sequence data arising from increasingly sophisticated sequencing technologies, including sequences from thousands of completed genomes, have sharpened these insights. In parallel, structure prediction has seen some quantum leaps over the last decade including from exploitation of AI and deep learning strategies that may bring structural annotations to many mysterious regions of sequence space currently uncharacterised. In this review we highlight some of the major shifts in technology and data that have enabled better exploration of protein structure space and brought functional insights.

### Early Identification of Protein Families

The technical challenges of determining 3D structures of proteins has meant that the sequence data has always outstripped structural data–currently more than 300-fold. There are approximately 170,000 protein structures in the PDB (Armstrong et al., 2019) but more than 200 million sequences in UniProt (The UniProt Consortium, 2019), and metagenomic data adds billions more sequences (Mitchell et al., 2019). In the late 70s and early 80s, Dayhoff pioneered the comparison of protein sequences, designing residue substitution matrices which enabled the alignment of even relatively distant relatives diverged from a common ancestor. Many other approaches have been explored since then (e.g. BLOSUM (Henikoff and Henikoff, 1992)), see review for others (Jones et al., 1992)). These approaches and the dynamic programming algorithms (e.g. developed by Needleman and Wunsch (Needleman and Wunsch, 1970), Smith and Waterman (Smith and Waterman, 1981)) developed to align protein sequences started the identification of protein evolutionary families by Dayhoff and others.

### How Constrained Are Protein Structures?

**FIGURE 1:**
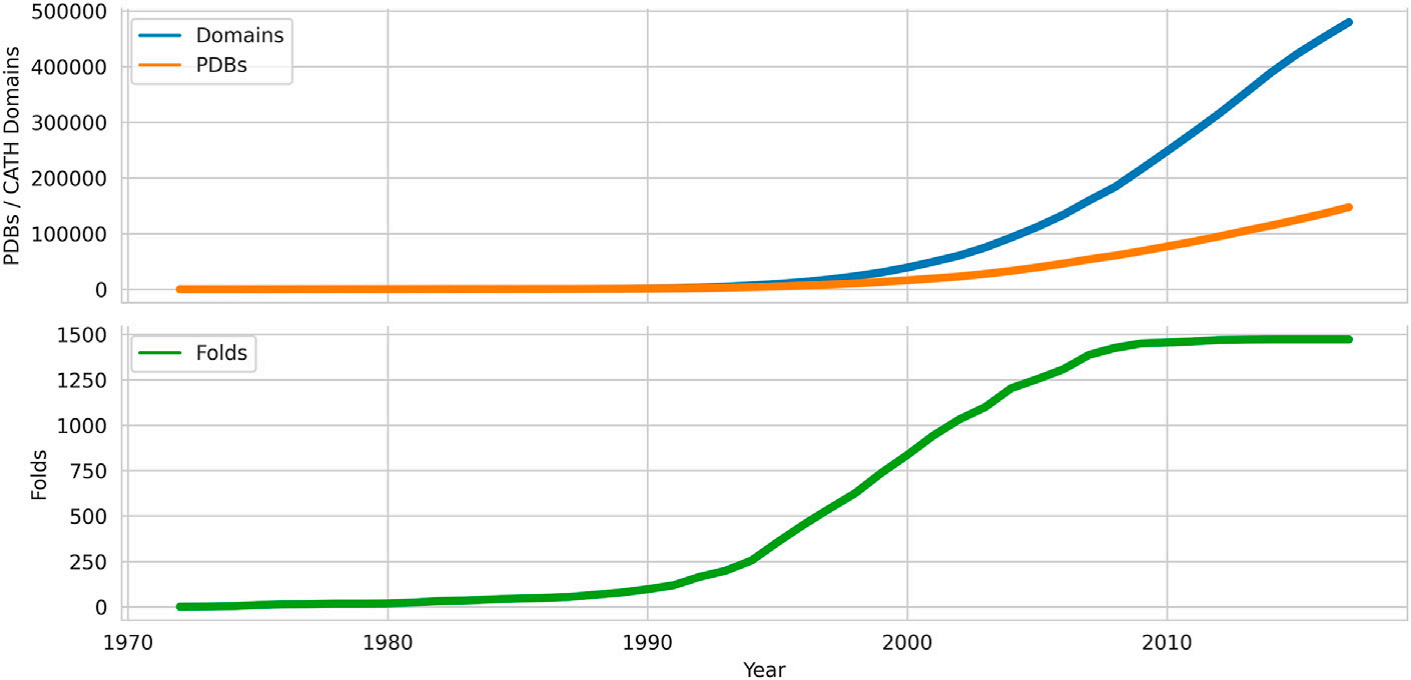
Growth of domains, folds and chains deposited in the Protein Data Bank from 1972 onwards. Data sources: PDB, CATH.

Adding structural data can help probe functional mechanisms more deeply and as the Protein Databank grew from the 1970s onwards (see Figure 1), algorithms for comparing structures emerged e.g. the still widely used rigid body approaches developed by Rossman and Argos (Rossmann and Argos, 1976) amongst others 9). As the PDB data grew it became clear that in some evolutionary superfamilies considerable divergence outside the structural core could occur.

**FIGURE 2:**
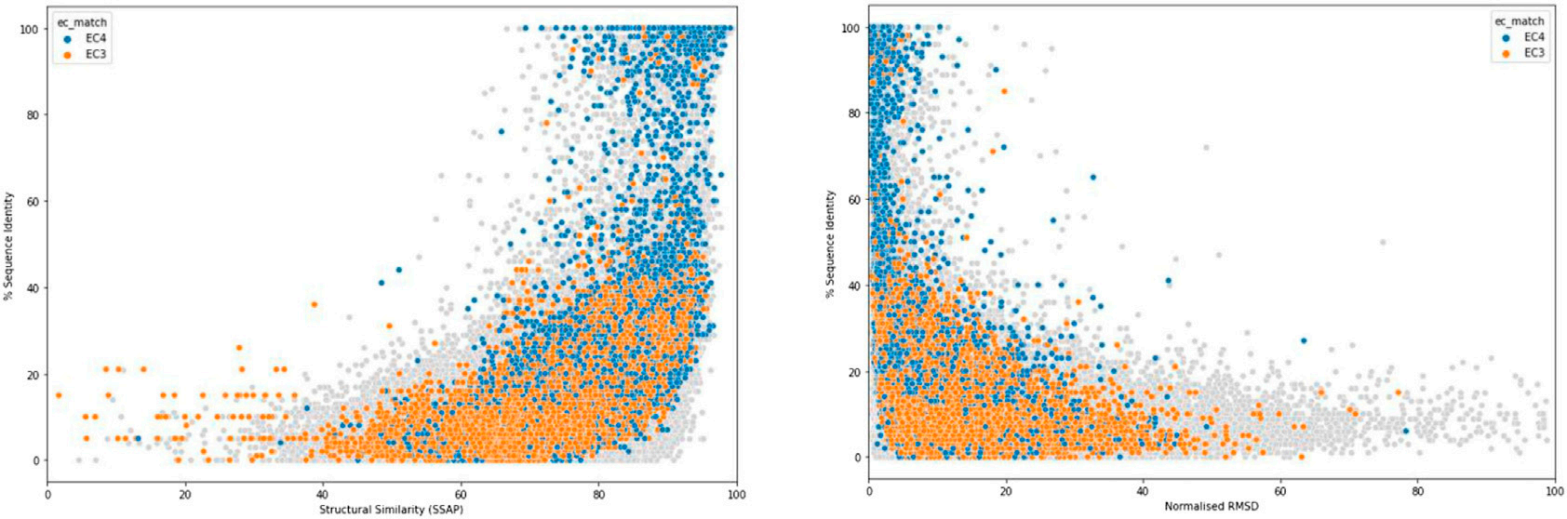
Structural similarity measured by SSAP score (left) or normalised RMSD (right) vs % of sequence identity.

One of the earliest and most important insights into structural divergence was captured by Cyrus Chothia and Arthur Lesk in their comparison of more than 32 pairs of protein homologues (Chothia and Lesk, 1986). This analysis showed the exponential relationship between sequence change and structural change and many of the characteristics captured in that study still hold when much larger datasets are examined. Figure 2 shows the relationship detected for current data using the SSAP structure comparison algorithm (see below and (Orengo and Taylor, 1996)). For relatives having similar functional properties, the structure is highly conserved even at low sequence similarity. Extreme divergence occurs for relatives with different functional properties, likely to be paralogues, having different structural constraints imposed by these functions.

To expand on these insights, Chothia and Lesk published some detailed and beautifully described expositions of the sequence structure relationships for two important protein families the globins (Lesk and Chothia, 1980) and the immunoglobulins (Chothia and Lesk, 1982; Lesk and Chothia, 1982).

To capture structural properties between very diverse homologues, many new methods emerged to better cope with the extensive residue mutations, insertions and deletions occurring between them. These methods have continued to evolve since the late 1980s. Many built on the dynamic programming strategies successfully exploited in sequence comparison. In some, dynamic programming was applied at two levels to fully exploit the 3D data. First at a low level (i.e. residue views) and then to an upper summary level to obtain the final alignment (e.g. see SSAP (Orengo and Taylor, 1996)). Other approaches combined rigid body superposition with dynamic programming (see for example early approaches STAMP (Russell and Barton, 1992), STRUCTAL (Subbiah et al., 1993), CE (Shindyalov and Bourne, 1998)). One of the most popular algorithms with crystallographers and other structural biologists, DALI (Holm and Sander, 1993), effectively “chopped” the structures into hexapeptide fragments and used Monte Carlo optimization to determine the optimal order for concatenating matched fragments between the structures. Other approaches commonly used by structural biologists include MAMMOTH (Ortiz et al., 2002) and GESAMT (Krissinel, 2012). Fast approaches (e.g. CATHedral (Redfern et al., 2007)) were also developed that explicitly compared secondary structure elements between proteins giving up to 1000-fold speedups in the alignments but at the cost of accurate residue alignments. These approaches were driven by the exponential increase in the number of structures in the PDB and the need for rapid scans with newly solved structures to identify novel folds. More recent approaches (e.g. FATCAT (Ye and Godzik, 2003)) have been explicitly designed to optimize the alignments between loops, typically the most diverse regions, but often containing key functional residues.

### Domain Based Structural Families

Chothia’s examination of the globins and immunoglobulins was the first step toward a more comprehensive analysis of structure space and analyses performed in the following decade culminated in the establishment of one of the most widely used resources capturing protein domain structure superfamilies–SCOP (Murzin et al., 1995) in 1994. SCOP was co-founded by Alexey Murzin, who joined Chothia’s team at the LMB and has remained a leading structure based evolutionary resource. Its first release contained 366 superfamilies, 866 non-redundant domain structures and 1182 protein domains from different species. As well as classifying domains by their superfamily, the superfamilies were also organized by class (determined by secondary structure composition) and fold group (determined by the order and orientation of those secondary structure elements in 3D space) in a hierarchical manner. Superfamilies in which relatives adopted regular arrangements in 3D were also annotated with architecture descriptions e.g. barrel, sandwich. Significant manual curation ensured very high quality in the assignments and annotations. SCOP has been expanded recently by inclusion of additional resources in SCOPe, managed by Steven Brenner and co-workers (Fox et al., 2014).

**FIGURE 3:**
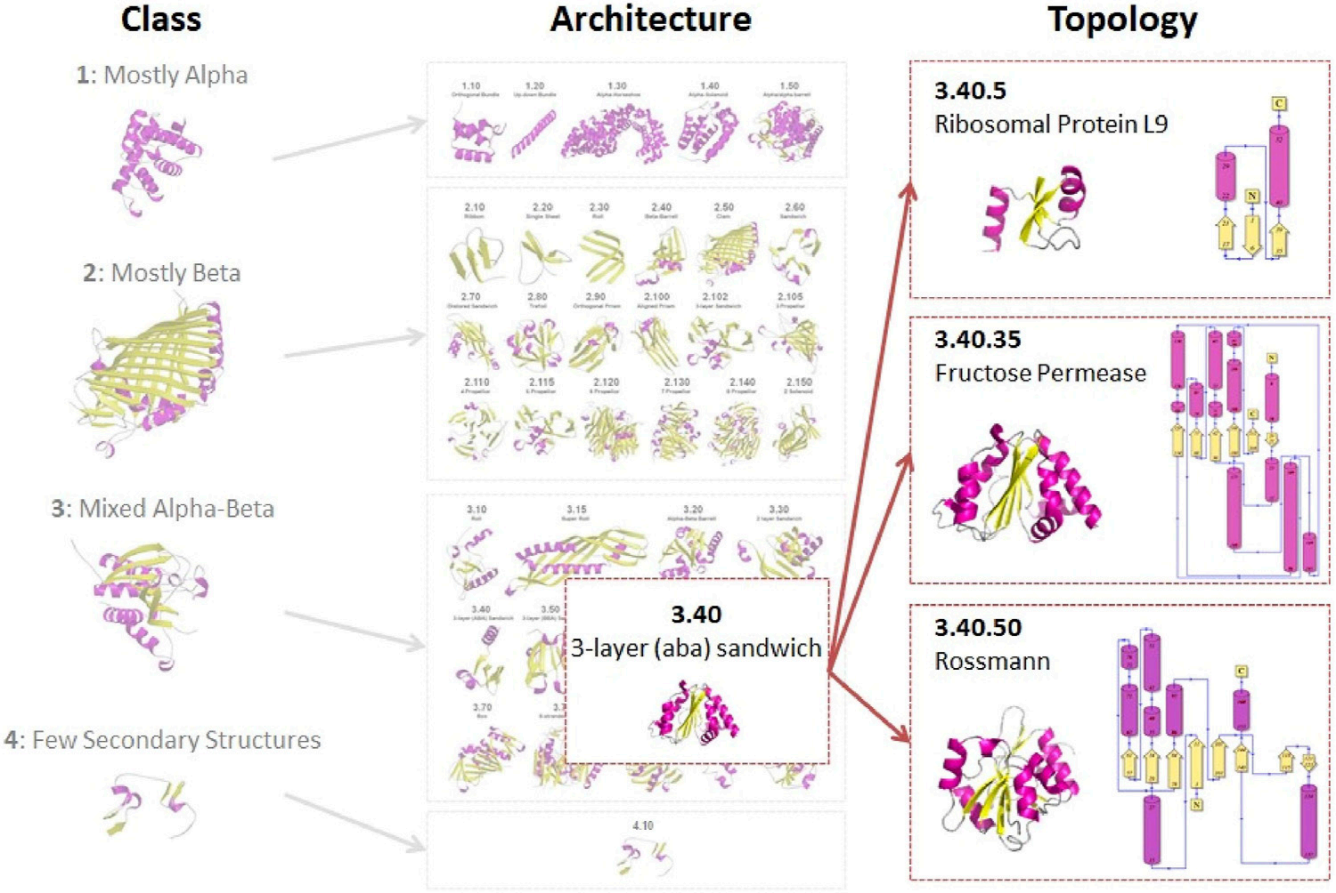
Overview of the CATH classification scheme for protein domains.

Continued expansion of the PDB has led to nearly a 10-fold increase in the number of superfamilies but the growth in new folds has been much slower (see Figure 1 for numbers from a related resource). In parallel, Janet Thornton’s group used a more automated approach by applying the SSAP structure comparison method (Orengo and Taylor, 1996), developed by Orengo and Taylor, to recognise homologues, including very distant homologues, and structures with similar folds. For extremely distant relatives, manual curation was also required but overall was not applied to the same extent as in SCOP. The CATH resource, set up by Orengo and Thornton, included a more formal architecture level within the hierarchy (see Figure 3).

**FIGURE 4:**
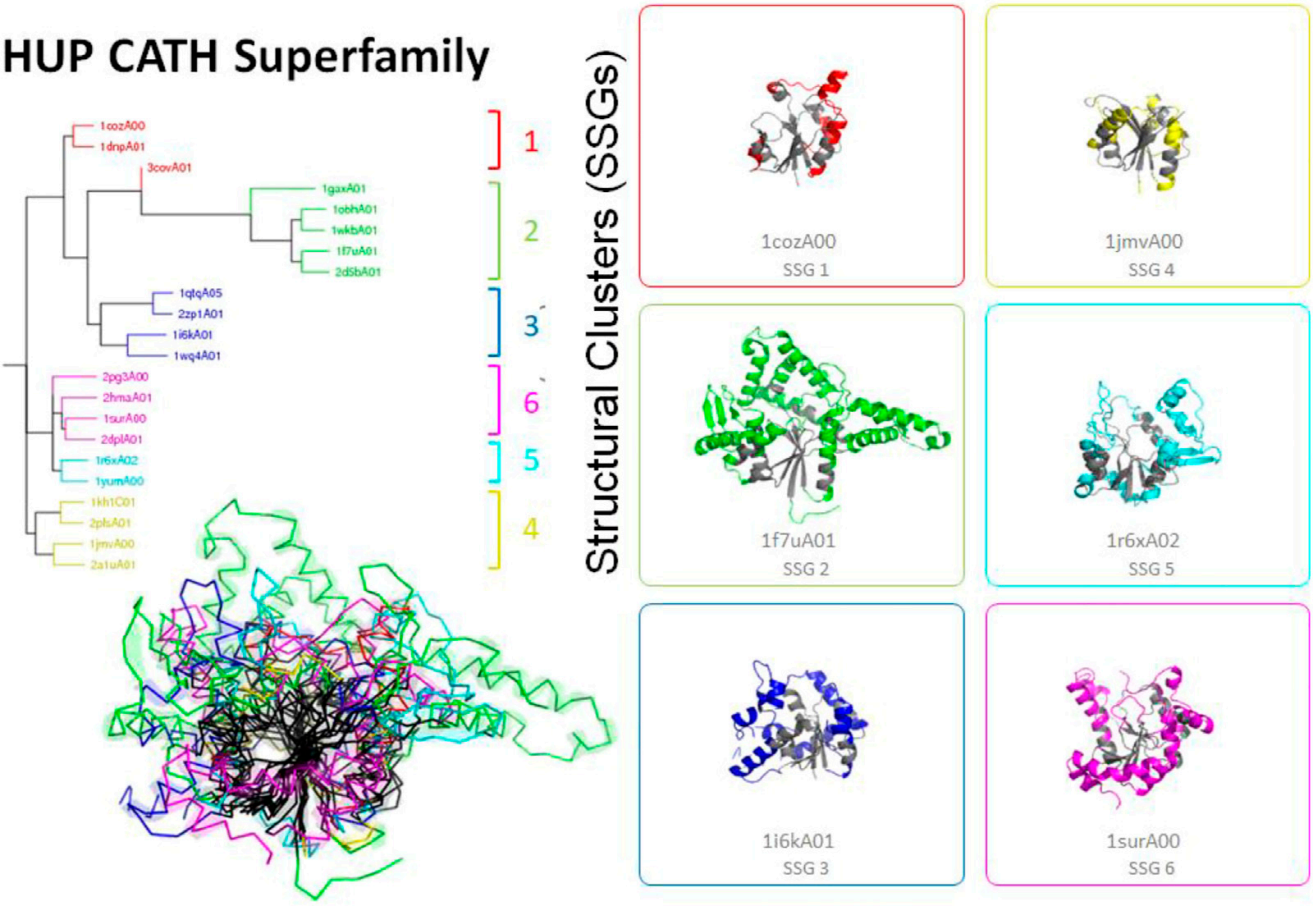
Highly divergent structural homologues within the HUPs SuperFamily (CATH ID 3.40.50.620). Six diverse structural clusters (also called structurally similar groups, SSGs) are identified using SSAP to compare structures all against all (see tree top left and figures on the right). However, representatives from each SSG can be superposed to reveal the highly conserved structural core common to all (see central black region in the bottom left figure).

**FIGURE 5:**
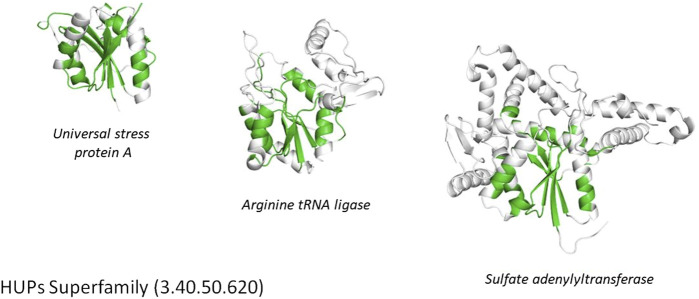
Conservation of the structural core (highlighted in green) within the HUPs superfamily.

As a result of the comparative ease of acquiring experimental data, the sequence databases (e.g. UniProt) expanded even more rapidly than the structure databank (PDB) and the increase in this information and more powerful profile based sequence comparison strategies to harness it e.g. PSI-BLAST (Altschul et al., 1997), HMMer (Eddy, 1998), HHsearch (Söding, 2005) aided in the confirmation of homologues in which structures had diverged considerably (see Figure 4). By capturing these extremely remote homologies, it became clear that sometimes only the structural core was conserved (see also Figure 5) (Dessailly et al., 2010). The variation in size across some superfamilies suggested a structural continuum and was also referred to as the “Russian Doll Effect” (Swindells et al., 1998). Furthermore, it was clear that some folding arrangements consisted of multiple repeat motifs e. g alpha-beta, beta-beta, alpha-alpha. Andrei Lupas and other groups highlighted primitive motifs appearing in early life that seeded the emergence of more complex folds through duplication and gene fusion (Lupas et al., 2001). In fact, a large scale application of the DALI algorithm on all known structures in the PDB, by Liisa Holm, identified a small set of very highly populated so-called “attractor” motifs (e.g. αβ, β−β, αβ) that link structural superfamilies (Holm and Sander, 1996).

More detailed SCOP and CATH-based analyses have suggested the need for a less rigid hierarchy and recent structural classifications, such as the ECOD resource developed by Nick Grishin (Cheng et al., 2014), have adopted this approach. In ECOD, domain structures are grouped into superfamilies annotated by class and architecture information but relatives within the superfamilies can be described as adopting different folds.

Both SCOP and CATH have also changed since their inception to reflect these phenomena. In 2014, SCOP2 was released (Andreeva et al., 2014, 2) providing many valuable links between superfamilies sharing common structural motifs. Rare structural motifs are also identified, and biochemical features highlighted. CATH now describes the topology annotations (fold or T-level) for each superfamily as reflecting “core structural motifs” since large scale comparisons of relatives show that for the majority of homologous pairs at least 50% of the structure is conserved and the core topological motif is a helpful structural fossil revealing even the most distant relationships.

### Unique Fold and Superfolds

**FIGURE 6:**
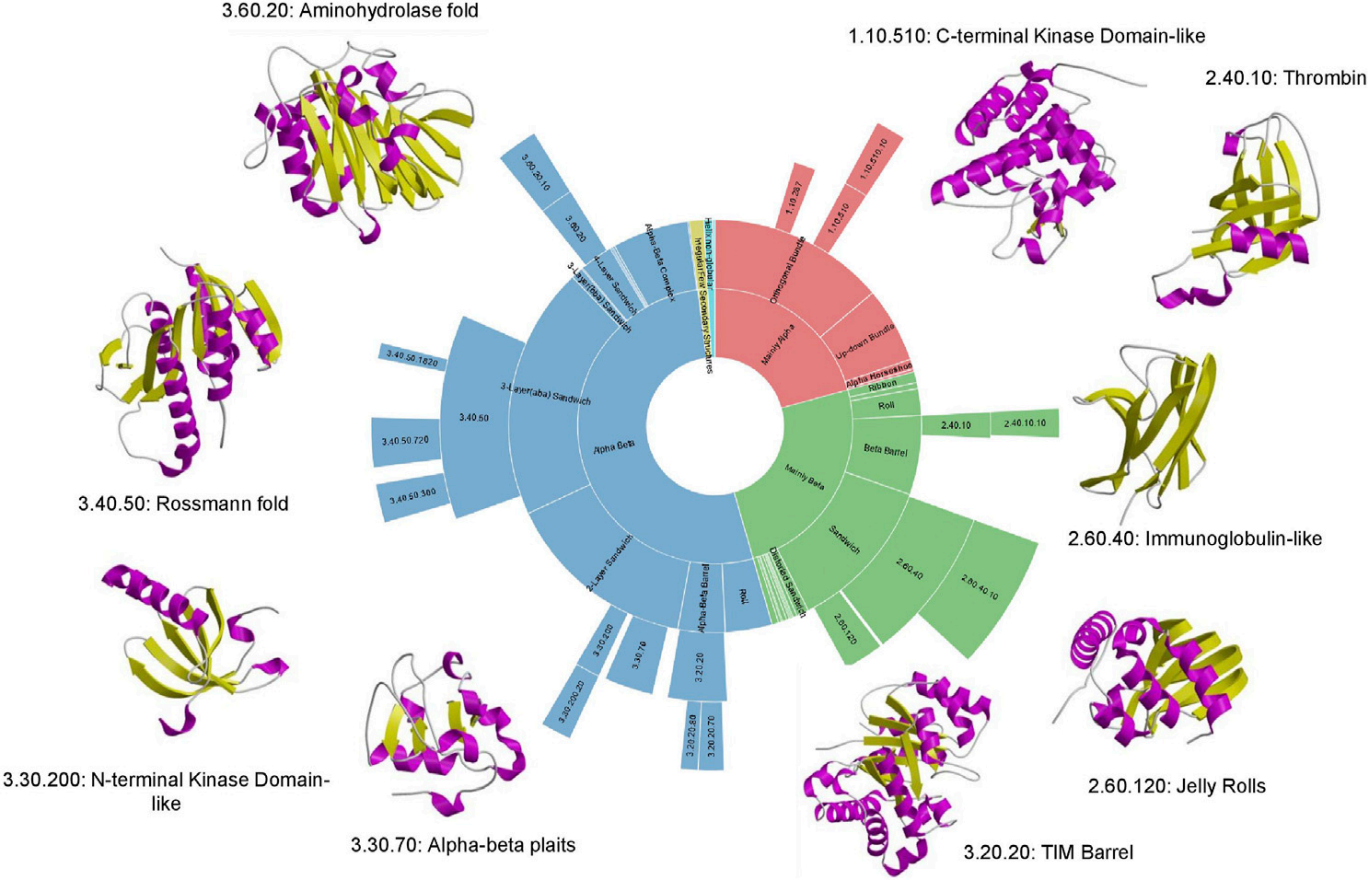
Top 9 “super-folds” in CATH v4.3. The inner wheel shows the proportion of structures that fall into each class, architecture, fold group and superfamily respectively.

Although structural classifications were clearly a valuable means of organising proteins and capturing evolutionary changes, a key question was the extent to which they reflected Nature or reflected bias in the Protein Data Bank. By using the more powerful profile-based sequence search strategies (e.g. PSI-BLAST) to map proteins with 3D-structures to all sequence relatives in UniProt, Chothia was able to show that even with the sparse structural data available at that time, a large proportion of sequences could be mapped to the SCOP families suggesting that these families were reasonably representative, though clearly they lacked many membrane associated proteins and disordered proteins (Chothia, 1992). That deficit still holds to some extent, although the PSI structural genomics initiatives which focused on membrane proteins helped to increase their representation in the structural classifications (Chandonia and Brenner, 2006). Current mapping done for some selected model organisms annotated in the integrated Genome3D resource ((Sillitoe et al., 2020) described below), shows that structural predictions based on SCOP or CATH superfamilies can be made for nearly 80% of proteins in many of these organisms, suggesting that a significant proportion of protein superfamilies in Nature are now represented in the protein structure classifications. In 1994, Cyrus Chothia made a prediction of fewer than 1000 folds in Nature (Chothia, 1992), an amazingly prescient estimation as 25 years later we possibly have as few as 1300–although there is still some controversy around the definition of fold! Furthermore, the dominance of some folding arrangements in Nature is still clear, with the top nine “superfolds” still accounting for more than 30% of all classified domain structures (see Figure 6). Of the current superfolds, five were detected in 1994 using CATH data (Orengo et al., 1994) and the remaining four superfolds (1.20.120, 1.10.490, 2.80.10, 3.10.20) were superseded by others (3.60.20, 2.40.10, 3.30.200, 1.10.510) that were less well populated in the original CATH release.

## Mapping Sequence Space to the Structural Families

While sequence to structure mapping has demonstrated that we have fold representatives for a large proportion of protein superfamilies in Nature, large parts of superfamily space are not yet covered by detailed structural and functional characterisation. This becomes even more apparent when metagenome sequence data is added e.g. from MGnify (Mitchell et al., 2019). Most structure classification resources make use of powerful tools like HMMer, developed by Sean Eddy and co-workers (Eddy, 1998) and HH-suite, developed by Johannes Soding to identify sequence relatives (Söding, 2005).

MGnify is 10-fold bigger than UniProt, currently comprising mostly prokaryotic data but the Earth Biome and Tree of Life sequencing projects (Lewin et al., 2018) will expand the data for eukaryotes too. The alpha-beta hydrolase superfamily, the 9th most populated superfamily in CATH (by number of non-redundant representatives at 90% sequence identity) is massively expanded (10-fold) by metagenome sequences extracted from a range of bacterial environments. Some of these e.g. from wastewater environments and oceans have changed in response to recent selection pressure leading to divergence in the binding site to accommodate PET and other plastics, which these enzymes can now degrade.

**FIGURE 7:**
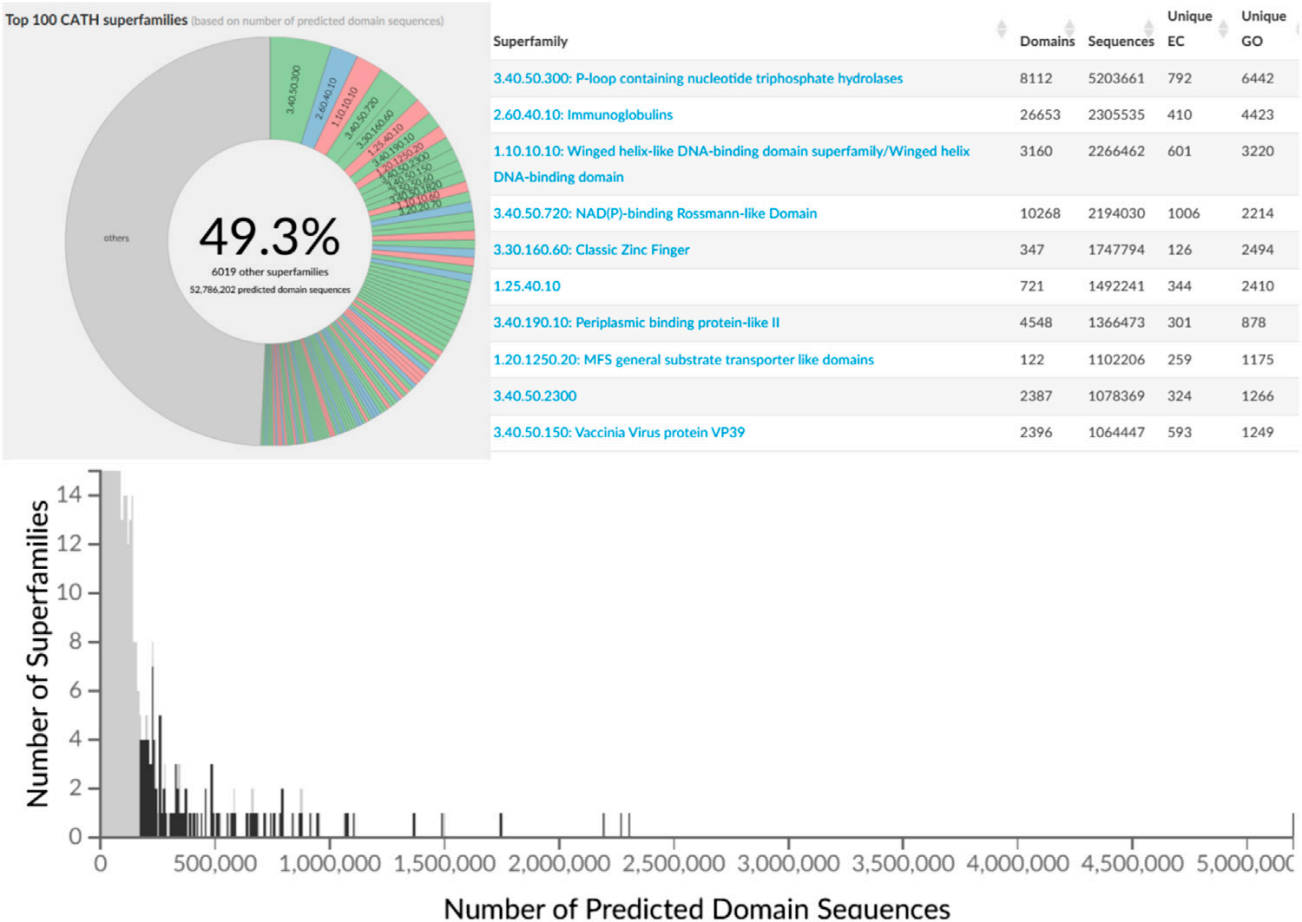
Top 100 most populated CATH SuperFamilies (CATH v4.3) with additional details regarding sequence counts and unique EC and GO terms for the top 10 most populated SuperFamilies.

The second phase of the PSI structural genomics in the States (2005–2010) explicitly targeted structurally uncharacterised protein sequences mapping to SCOP, CATH or Pfam superfamilies to extend structural knowledge of these dark regions of sequence space (Norvell and Berg, 2007). These analyses further confirmed early expositions of the power law in structure-sequence space whereby some superfamilies had been massively expanded through extensive gene duplication throughout evolution. Many of these very highly populated superfamilies (described as “Megafamilies” by the structural genomic initiatives), are universal to all kingdoms of life and contain domains performing essential generic functions, like the many Rossman superfamilies which bind nucleotide cofactors e.g. NAD or NADP, in a common cleft in the structure formed by a crossover in the polypeptide chain. Figure 7 shows that currently the top 100 superfamilies account for nearly 50% of all protein domain sequences mapped to CATH structure superfamilies.

## Protein Domains Are Combined in Millions of Different Ways in Nature

Analyses of the SCOP and CATH superfamilies confirmed the generic functional role of many domain relatives (see further discussion below) and the commonly used description of domains as independently folding functional units in evolution. The incredible enhancements in sequencing technologies at the turn of the millenium, allowing sequencing of whole genomes starting with human, meant that comparative genomics studies became possible exploring the different distribution of domain families and domain combinations within and between different kingdoms of life. There are now more than 300 complete and nearly complete genomes in ENSEMBL (Yates et al., 2020). This genomic data showed the extent of gene duplications, gene fusions and fissions occurring during evolution, with the former being more common (Björklund et al., 2005). Changes in these multidomain combinations or multidomain architectures (MDAs) result in expansions and divergence in the functional repertoires between species in response to selective pressures imposed by novel environmental contexts.

**FIGURE 8:**
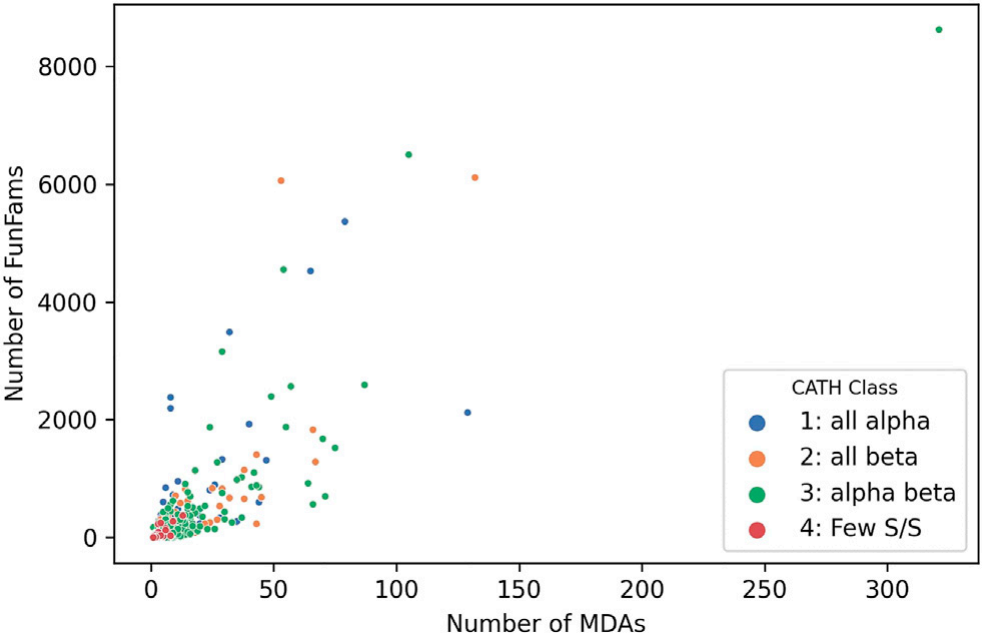
Number of MDAs vs Number of sequence subfamilies (FunFams) for each SuperFamily in CATH v4.3.

Studies inspired by Chothia’s vision of domain units taken forward by various researchers he mentored, notably Sarah Teichmann and Mark Gerstein, characterised the “mosaic” nature of proteins and confirmed domains as the fundamental building blocks of life (Teichmann et al., 1999; Teichmann et al., 2001). Analyses of CATH-Gene3D which contains domain sequences from UniProt mapped to CATH and Pfam superfamilies using HMMer-based protocols currently reveal 311,575 different domain combinations. This is probably an underestimate since many proteins have regions of sequence that are still uncharacterized and may correspond to novel families that are unlikely to be common to multiple species. Unsurprisingly the more sequence sub-families found within a superfamily the more multidomain architectures identified (see Figure 8, below) and the power law is apparent again with the top 100 superfamilies occurring in the most MDA contexts occurring in a very large number of different MDA contexts (51% of all). Changes in domain context can modify the active site or binding pockets (discussed more below) and inevitably alter the surface features of the protein enabling diversity in protein interactions for paralogs expressed in different tissues. In addition, Teichmann and co-workers showed that some combinations of domains, described as supradomains, are particularly prevalent, probably corresponding to useful functional units (Vogel et al., 2004).

Comparative genome studies enabled by this vast sequence data could probe deep evolutionary relationships by using the structural families identified for different species. For example, CATH-based studies showed essential pathways populated by universal superfamilies that can be traced back to the Last universal Common Ancestor (LUCA) (Ranea et al., 2006).

## Structure Families Bring Detailed Insights Into Protein Function Evolution

Chothia’s eloquent reviews of domain structure families and evolutionary changes in protein structures were inspiring and played a key role in framing the questions around protein function evolution. In particular, he sought to elaborate on a “domain grammar of function” that would allow translation of a multi-domain “sentence” based on the functional roles of the constituent domains.

Other complementary studies added to the emerging picture. For example, Thornton’s group analysed 31 highly populated and well structurally characterised superfamilies in CATH revealing the extent to which functions could diverge in particular in the megafamilies (Todd et al., 2001). A number of phenomena can drive this. Clearly the existence of multiple relatives in a genome means that extra copies (i.e. paralogues) will be more tolerant of mutations and these can drive functional shifts if they occur on or near key sites. In addition, as mentioned already, domain fusions can reshape functional sites or surfaces. Furthermore, relatives can oligomerise in different ways again driving structural modifications in the active site or functional surfaces and the creation of new surfaces capable of evolving functional roles.

However, dramatic changes in functional class or in the chemistry performed by an enzyme, for example, appear to be rare (Todd et al., 2001; Bashton and Chothia, 2007). It’s hard to engineer the geometry and exquisite stereospecificity needed to perform an enzyme reaction and perhaps not surprising that these analyses revealed a significant tendency for chemical intermediates to be conserved along the reaction pathways of different relatives in the superfamily. More frequently, evolutionary changes (particularly residue insertions) cause changes in the geometry of the active site and binding pocket enabling relatives to perform the same or similar chemistry on a different substrate (Todd et al., 2001).

These evolutionary changes, which can sometimes be quite subtle involving just a handful of residues, combined with the expansion of paralogs through gene duplication give an effective mechanism for expanding the functional repertoire of an organism. For example, the kinase superfamily has been significantly expanded in eukaryotes where relatives perform essential functions in cell-cell communication and intracellular signaling. Most paralogs are involved in phosphorylation of protein targets, but these targets can vary and relatives may be expressed in different tissues having diverse interaction opportunities.

**FIGURE 9:**
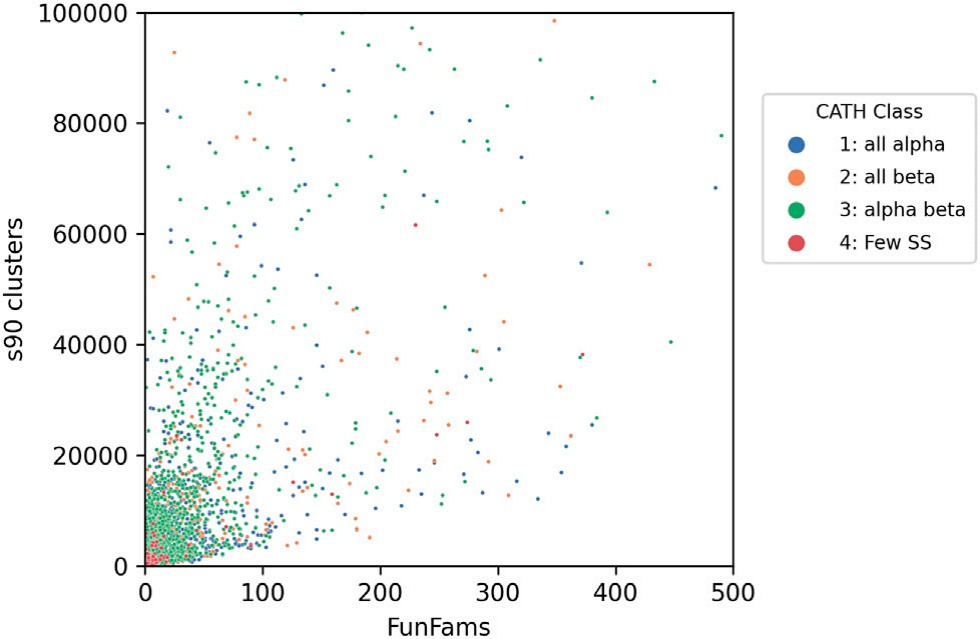
Functional diversity (captured by number of functional families - FunFams) vs. sequence diversity (number of Gene3D s90 clusters i.e. in which relatives share 90% or more sequence identity) for CATH superfamilies. Each dot represents an individual superfamily.

Bashton and Chothia (Bashton and Chothia, 2007) undertook a very detailed analysis of the extent to which key functional roles were conserved across domain superfamilies allowing domains to be used as “words” within a protein “functional sentence”. This is a challenging task, and the challenges increasingly apparent as more experimentally characterised sequence relatives are classified within SCOP and CATH. In SCOP these predicted structural relatives are classified in the sister resource, SUPERFAMILY, managed by Julian Gough (Wilson et al., 2009). In CATH, sequences are directly integrated in superfamilies as well as being captured in the Gene3D sister resource (Lewis et al., 2018). Currently, the sequence data from UniProt expands the structural superfamilies 500-fold on average (up to 49 thousand-fold), depending on the superfamily allowing a deeper analysis of functional diversity. The correlation between sequence diversity and the number of sequence subfamilies and functional diversity can be seen for all types of superfamilies in Figure 9.

Chothia’s analyses supported earlier hypotheses of conservation of function within a broad functional class (Bashton and Chothia, 2007). For example, the amino-acyl tRNA synthase superfamily is amongst the top 2% largest superfamiles and relatives perform multiple functions covering at least 31 EC3 categories (i.e. having different EC classifications at the third EC level associated with change in chemistry). Nevertheless, many relatives exploit the same co-factor pyridoxal 5-pyrophosphate binding to the same site and substrates tend to share a similar chemical moiety.

Another functionally diverse superfamily, the HUP superfamily, currently contains more than 640 thousand sequences from UniProt and 39,505 sequence subfamilies (at 50% sequence identity). This threshold is used because various studies have suggested 50% or 60% sequence identity for inferring functional similarity between homologues, provided there is reasonable overlap in sequence length (60% or more) (Rost, 2002; Rentzsch and Orengo, 2009). CATH identifies at least 55 EC terms and 594 diverse Gene Ontology (GO) terms for experimentally characterized relatives within this superfamily and like many other megafamilies less than 6% of relatives have experimental characterization. In addition, it’s possible to characterize the structural diversity across this superfamily by clustering relatives according to structural similarity (e.g. < 5 A RMSD). There are currently 31 such structural clusters. Despite this structural and functional diversity the structural core is highly conserved (see Figure 5 above), as also observed in other megafamilies. However, as seen in Figure 4, there can be considerable structural decorations or embellishments outside this core.

### Phylogenetic Insights

The vast sequence data available for many species has allowed phylogenetic forays into protein superfamilies. For example, by combining both structural and sequence data as in CATH-Gene3D we can trace further back and explore the order of functional shifts within these superfamilies. The FunTree classification studies of Thornton and co-workers allowed tracing of shifts in enzyme chemistry (changes in the third number of the EC classification code) between homologues in all highly populated superfamilies in CATH (Furnham et al., 2012).

Similarly expansion in the structural data available for the superfamilies, thanks partly to targeted activities of the PSI structural genomics initiatives, provided insights into shifts in catalytic residues within enzyme superfamilies (Todd et al., 2005), confirming trends detected by early studies of Thornton and co-workers using much sparser data. As in the previous analysis interesting cases of convergence of catalytic machinery within superfamilies or “residue hopping” were detected (Todd et al., 2002). This was caused by divergence of functionally distinct homologues which then converged to the same chemistry via different mutational routes giving catalytic residues in different places in the active site pocket, but with the same chemical properties and necessary orientation to perform the chemistry.

## Functional Sub-Classification of Protein Reveals the Dark Matter of Function Space

With <1% of protein sequences in UniProt having experimental characterisation, interest has grown in understanding the likely functional divergence across superfamilies, especially those with industrial value. Organising the sequence data to reveal highly conserved residues between putative functional relatives can give clues to possible changes in substrate specificity or enzyme chemistry. Because the structural data is so sparse, our approach to identifying functional families (FunFams) in CATH superfamilies has been to use sequence data and cluster relatives using an entropy-based method that segregates sets of relatives with differentially conserved residues (Das et al., 2015b). Residues that are conserved across all relatives in a superfamily are likely to be important for folding or stability but residues that are conserved in different ways e.g. residues with different chemical properties, between different sets of relatives, are likely to be associated with the functional roles of the proteins. Some endorsement of this functional clustering is given by performance of CATH functional families in the independent CAFA Critical Assessment of Functional Annotations (Jiang et al., 2016; Zhou et al., 2019). Furthermore, residue sites conserved in FunFams are significantly enriched in known functional residues e.g. catalytic residues, protein interface residues, ligand binding residues etc (Das et al., 2015b).

**FIGURE 10:**
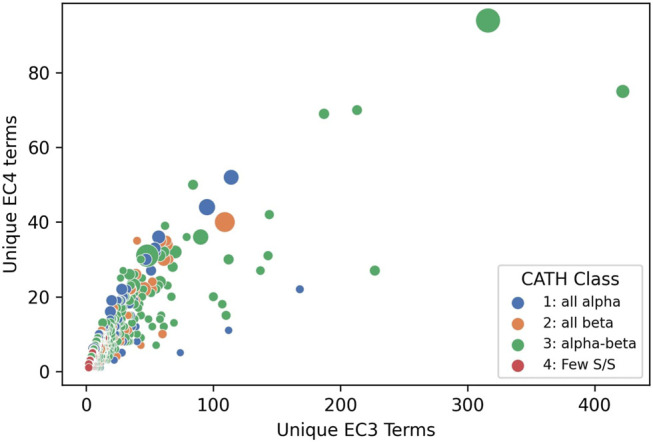
Enzyme Commission terms distributions for each CATH v4.3 SuperFamilies, showing that 65 superfamilies have more than 20 different chemistries (i.e. EC3s).

Structural data, whether known or predicted, can then be exploited to determine where these putative functional determinants co-locate on the protein surface to glean further insights into functional properties. This clustering into functional families reveals the most promiscuous, highly diverse superfamilies. Figure 10 shows that the top 65 most functionally diverse enzyme superfamilies have more than 20 different chemistries exhibited by relatives.

FunFams are only identified for sets of sequences where at least one relative has been experimentally characterized and has a GO functional annotation. On that basis, only about 36% of the 150 million domain sequences classified in CATH can be assigned to a functional family suggesting that there is still a large proportion of functional space to characterize. However, some superfamilies, particularly those containing important eukaryotic organisms (e.g. human, model organisms) tend to have a higher proportion of functional characterization. It’s also important to remember that this is a domain based functional classification, but function is generally annotated at the protein level. However, analyses of selected superfamilies, namely the enolases, TPPs and HUPs suggest that by segregating on functional discriminants domain relatives occurring in different multidomain contexts are indeed clustered into separate functional groups (Das et al., 2015a).

## Functional Families Give Finer Insights Into the Emergence of Novel Functions in Metazoa

As mentioned already above, globular domains are one of the key functional units of proteins, often with a specific functional role and with the ability to fold independently. Some globular domains have catalytic functions, facilitating enzymatic reactions, providing much of the complex chemistry that cells need to function. Other domains are responsible for detecting signals, by interacting with other protein domains and ligands, as part of signaling processes.

**FIGURE 11:**
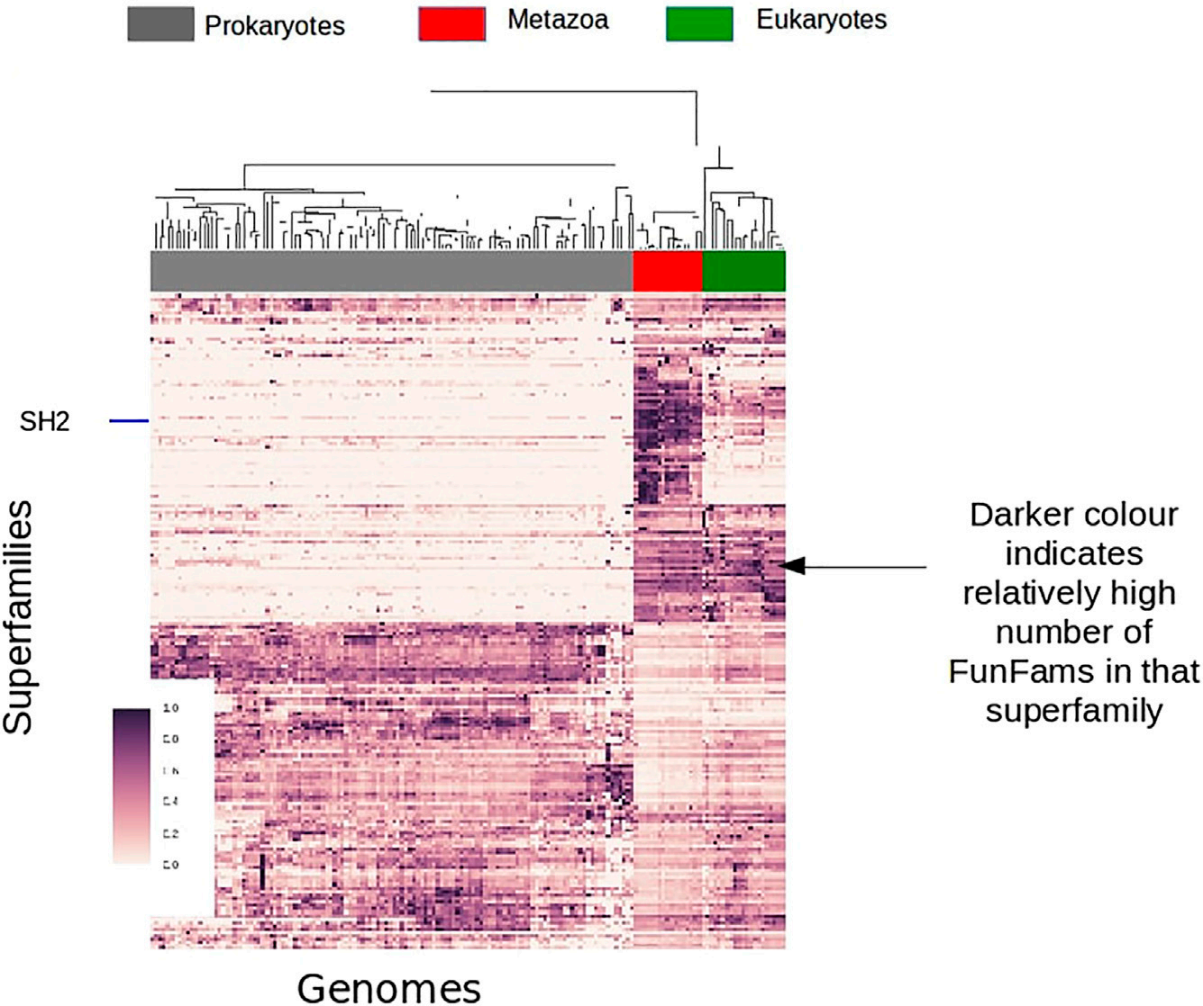
Survey of FunFam expansions across the tree of life, darker colors show a higher number of FunFams in that superfamily.

During the history of life on Earth there have been a number of major evolutionary events each requiring their own unique functional innovations. For example, early life-forms needed to establish much of the initial basic chemistry, energy production, metabolism etc. A number of domain superfamilies date back to the last universal common ancestor (LUCA) of cellular life, and these domain superfamilies provide much of the catalytic processes required by cells, such as the TIM-barrel domain superfamily, which provides the basic structural core for hundreds of different catalytic functions. Another major transition was the emergence of animals (Metazoans), which appeared several hundred million years ago, from single-celled ancestors (Figure 11). The emergence of Metazoans required many different functional innovations relating to cell communication, differentiation and migration. To support cellular complexity, coordinated regulation of gene expression was needed together with many other protein innovations such as the establishment of various signal transduction pathways that connect extracellular signals to transcriptional regulation.

As already mentioned, gene duplication and fusion can give proteins with novel domain combinations leading to new functions. For example, changing the multi-domain architecture, can give a novel protein that operates in a new cellular micro-environment. However, a change in multi-domain architecture is not a prerequisite for domain-based innovations and domains may gain novel functions with no change in domain partners. We can use a change in CATH FunFams, as a proxy for a change in domain function, allowing a preliminary exploration of various aspects of Metazoan evolution from a FunFam domain perspective.

For example, by examining the expansion in the number of FunFams within a domain superfamily we can track the expansion of functional diversity across that superfamily at different stages in evolution. By counting the number of FunFams for a given superfamily and clustering organisms using TreeFam (Ruan et al., 2007) we can show the FunFam expansions in CATH superfamilies at different evolutionary stages.

A large number (of domain superfamilies) show strong expansions (in the number of their FunFams) specifically at the emergence of Metazoans. Many of these expanded superfamilies are associated with signaling and regulatory processes, such as the SH2 domain family which undergoes significant expansion in Metazoans corresponding to its newly acquired role of phosphotyrosine binding domain in cell signaling processes. Transcription factors are known to have a key role in Metazoan evolution/development. Many Transcription factor FunFams appear early in Metazoan evolution, prior to the separation of extant metazoan phyla but after the divergence of Choanoflagellates and Metazoans. There is also further lineage specific expansions in transcription factors, for example along the vertebrate lineage.

## Conclusion

The pioneering work of Cyrus Chothia in characterising the relationship between sequence and structure and his subsequent analyses of specific families, namely the globins and immunoglobulins, together with structural and functional analyses by Janet Thornton amongst others, inspired algorithms and analytic protocols for detecting evolutionary relationships and the mechanisms by which genetic variations translate into structural and functional changes during evolution. These frameworks provided impetus for the establishment of comprehensive structural classifications which have been exploited in many analyses shedding light on divergence, particularly for enzyme superfamilies, but which also established general principles regarding functional shifts in all protein classes. To some extent the SCOP and CATH classifications have provided complementary perspectives as the former involved detailed manual curation and explicitly recognised domains found in diverse multi-domain contexts. In contrast, CATH aimed to exploit computational strategies that searched for globular domains and then classified them based on structural similarities in the core. Unlike many fields of science where competition often clouds judgment, Cyrus was a man of huge intellect and integrity who valued competition and the opportunities that diverse perspectives give in maximising the exploration and understanding of complex phenomena. He was one of the most supportive scientists in the Genome3D consortium which established formal collaborations between SCOP and CATH and which is currently enhancing the structural coverage of genome sequences in human, model organisms and Pfam families (Sillitoe et al., 2020). This collaboration is being continued in the new 3D-SCAfold initiative, being led by PDBe, which will ensure closer integration and disseminate the family data more widely to enable deeper studies of evolution.
